# Circulating MiR-374a-5p is a potential modulator of the inflammatory process in obesity

**DOI:** 10.1038/s41598-018-26065-5

**Published:** 2018-05-16

**Authors:** Ayo P. Doumatey, William J. He, Amadou Gaye, Lin Lei, Jie Zhou, Gary H. Gibbons, Adebowale Adeyemo, Charles N. Rotimi

**Affiliations:** 1 Metabolic, Cardiovascular and Inflammatory Disease Genomics Branch, National Human Genome Research Institute, Bethesda, MD USA; 2 The Center for Research on Genomics and Global Health, National Human Genome Research Institute, National Institutes of Health, Bethesda, Maryland USA; 3 Johns Hopkins University, Krieger School of Arts and Sciences, Baltimore, Maryland USA; 4 National Heart, Lung, and Blood Institute, National Institutes of Health, Bethesda, MD USA

## Abstract

Obese individuals without expected metabolic co-morbidities are referred to as metabolically healthy obese (MHO). The molecular mechanisms underlying this phenotype remain elusive. MicroRNAs may be involved in the MHO phenotype. To test this hypothesis, we screened 179 serum miRNAs in 20 African-American women (10 MHOs and 10 metabolically abnormal obese individuals -MAO). We identified 8 differentially expressed miRNAs (DEMs) with validation in an independent sample of 64 MHO and 34 MAO. Of the eight DEMs in the screening phase (p ≤ 0.05), miR-374a-5p remained significant (p = 0.04) with directional consistency in the validation sample. Ingenuity Pathway analysis revealed that miR-374a-5p putatively targeted 37 mRNAs (e.g. chemokines and transcription factors) which are members of canonical pathways involved in inflammation (IL-17A signaling) and lipid metabolism. Analysis restricted to adipocytes, the main source of circulating miRNAs in obesity, identified 3 mRNAs (CCL2, STEAP2, EN1) as the main target of miR-374a-5p. Evaluation of the 3 mRNAs in an independent sample showed that CCL2 was significantly downregulated (p = 0.0005). In summary, MiR-374a-5p is upregulated in MHO compared to MAO individuals and appears to show association with downregulation of pro-inflammatory markers that are linked to insulin resistance. Given the correlative nature of our findings, functional studies are needed.

## Introduction

Obesity is characterised by excessive fat accumulation that is mostly the result of excess energy intake relative to levels of physical activity^1^. However, the development of obesity is also influenced by lifestyle-independent factors like genetic background and hormone dysfunction^1^. Obesity has been shown to alter metabolic function. At the onset of obesity, adipose tissue expands through either hypertrophic expansion, which occurs when the volume of pre-existing fat cells is increased, or hyperplasia, which occurs when new small fat cells are generated^2^. Hypertrophic adipose tissue has been shown to be highly correlated with metabolic complications compared to hyperplastic adipose tissue^3^. Also, excessive abdominal fat impacts metabolic health more adversely than subcutaneous fat^4^. Furthermore, obesity is a chronic low-grade inflammatory disease state that contributes to insulin resistance (IR), type 2 diabetes (T2D), and cardiovascular disease^5^.

While most obese persons display metabolic risk factors such as high blood pressure, hyperglycaemia and dyslipidaemia and are thus classified as metabolically abnormal individuals with obesity (MAO), a small subset of obese individuals appears to be metabolically healthy. These persons are referred to as metabolically healthy individuals with obesity (MHO)^6^. While the concept of MHO is clear, there is a lack of consensus regarding the criteria used for the definition of the MHO phenotype. This has resulted in a wide variation in the reported prevalence, depending on the stringency of the definition used. A recent review by Rey-Lopez *et al*., reports MHO prevalence ranging from as low as 6% to as high as 75%^7^. There is accumulating evidence suggesting that the MHO phenotype may represent a transitional physiologic state in which obese individuals are not protected from metabolic consequences later in life^8,9^. However, it has also been shown that a subset of healthy obese individuals remain healthy into their elderly years, as MHO do not have a higher risk of all-cause mortality when compared to their normal weight counterparts^10^. Understanding this subgroup of obesity at the molecular and cellular level may help decipher the pathophysiology of the occurrence of obesity related co-morbidities. Thus far, few studies have investigated the molecular basis of MHO in human populations^11–13^.

Micro-ribonucleic acids (miRNAs) are key gene regulators that are potential candidates as molecular mediators of many human disorders. Indeed, studies have shown that miRNAs play an important role in many cardio-metabolic diseases, such as obesity, type 2 diabetes, atherosclerosis, and metabolic syndrome^14–17^ but the importance of miRNAs in MHO has not been investigated. MicroRNAs (miRNAs) are short, highly conserved RNAs that are used in post-transcriptional gene regulation by negatively altering gene expression through cleavage of mRNA or inhibition of protein translation^18^. MiRNAs are extremely important in gene regulation, as only a few hundred miRNAs are estimated to regulate 30–80% of the genes in the human genome, with each miRNA being capable of targeting hundreds of genes, and each gene having the capacity to be targeted by multiple miRNAs^19^.

Since MHO individuals maintain their metabolic health despite excess fat mass, they represent a key subset in understanding the role of miRNAs in regulating metabolic functions in obese individuals. Also, because MHO individuals are suspected of being in a transitional phase between healthy normal weight and MAO^8,9^, understanding the differences between the miRNA profiles of MHO and MAO can shed light on the importance of miRNA regulation in the development of obesity-associated metabolic disorders. In this study, we investigated the serum expression levels of a panel of 179 miRNAs to determine differential expression patterns in the miRNA profile of MHO and MAO individuals.

## Results

### Demographic and clinical characteristics of the screening cohort

The screening phase of the study included 20 African American (AA) women, 10 with MHO and 10 with MAO. As expected, MHO group had a more favourable metabolic profile compare to the MAO group. Their lipid, inflammatory and glycolytic profiles were in the normal ranges whereas MAO group had higher triglycerides/high density lipoprotein-cholesterol (TG/HDL-C) ratio and C-reactive protein (CRP) compared to the MHO group. There were no significant differences in age and body mass index (BMI) between the two groups (p > 0.05) but waist-to-hip ratio (WHR), a measure of abdominal obesity, was significantly higher in MAO than MHO (p_WHR_ = 0.004) (Table 1).

**Table 1:** Anthropometric and clinical characteristics of participants included in the screening phase of the study.

Variable	Overall (n = 20)		MHO (n = 10)		MAO (n = 10)		P-value^¥^
	Mean (Std. Dev)	Range (Min - Max)	Mean (Std. Dev)	Range (Min - Max)	Mean (Std. Dev)	Range (Min - Max)	
AGE (Years)	42.30 (8.85)	30.00–59.00	41.30 (10.08)	30.00–58.00	43.30 (7.85)	31.00–59.00	0.6265
BMI (Kg/m^2^)	40.62 (10.62)	31.13–70.32	36.49 (5.69)	31.16–48.67	44.75 (12.95)	31.13–70.32	0.0892
WHR	0.86 (0.07)	0.71–0.98	0.82 (0.06)	0.71–0.91	0.90 (0.05)	0.84–0.98	0.0042*
SBPS (mmHg)	131.98 (22.74)	105.00–195.00	118.20 (6.32)	105.00–125.50	145.75 (25.11)	113.00–195.00	0.0071*
DBPS (mmHg)	80.08 (11.87)	67.00–109.00	73.75 (4.78)	67.00–81.00	86.40 (13.63)	67.50–109.00	0.018*
Glucose (mg/dl)	93.18 (15.33)	78.00–124.50	83.35 (4.50)	78.00–91.00	103.00 (16.17)	81.50–124.50	0.0038*
Log10HOMA-IR	0.65 (0.52)	−0.09–1.93	0.22 (0.22)	−0.09–0.51	1.08 (0.32)	0.83–1.93	<0.0001*
Geometric mean	**4**.**47**		**1**.**66**		**12**.**09**		
TG/HDL-C	2.26 (3.87)	0.39–16.95	0.59 (0.25)	0.39–1.18	3.92 (5.04)	0.93–16.95	0.066
Log_10_CRP(mg/dl)	−0.32 (0.63)	−1.22–0.68	−0.86 (0.24)	−1.22–0.54	0.22 (0.37)	−0.40–0.68	<0.0001*
Geometric mean	**0**.**48**		**0**.**14**		**1**.**66**		

^¥^Student’s t-test p-values comparing mean between MHO and MAO.

^*^Significant p-value (p < 0.05).

### Identification of differentially expressed miRNAs in the screening phase and validation in an independent cohort

**Figure 1:**
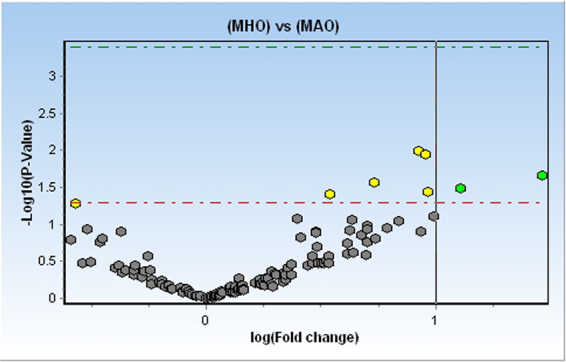
Volcano plot representing differentially expressed miRNA in the screening phase. Green dots represent miRNAs with FC > 2 and p < 0.05; Yellow dots represent miRNAs with FC < 2 and p ≤ 0.05. Gray dots represent miRNAs not differentially expressed between MHO and MAO.

**Figure 2:**
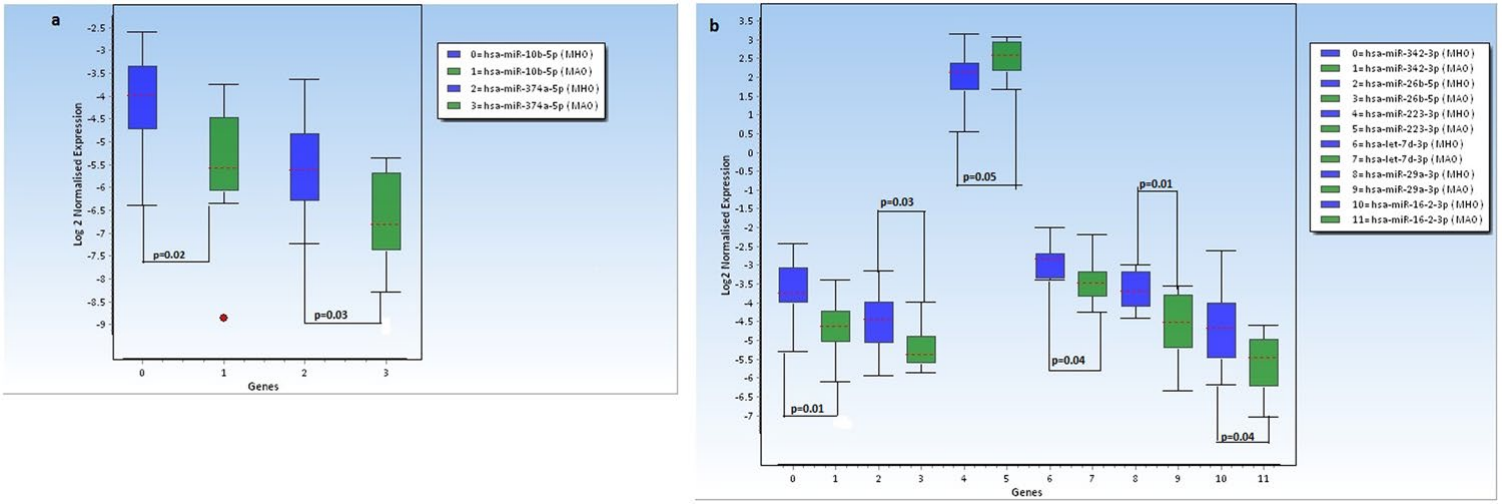
Box Plots depicting differentially expressed miRNAs in MHO compared to MAO (screening phase). (**a**) Two most differentially expressed miRNA in MHO compared to MAO (FC > 2). (**b**) Six DEMs (FC < 2, p <= 0.05).

We identified 8 differentially expressed miRNAs (DEMs) between MHO and MAO (p <= 0.05); Seven were upregulated including miR-10b-5p (FC = 2.75, p = 0.02) and miR-374a-5p (FC = 2.15, p = 0.03) (Fig. 1, Fig. 2) and one miRNA was downregulated in MHO compared to MAO (miR-223-3p, FC = −1.48, p = 0.05).

**Figure 3:**
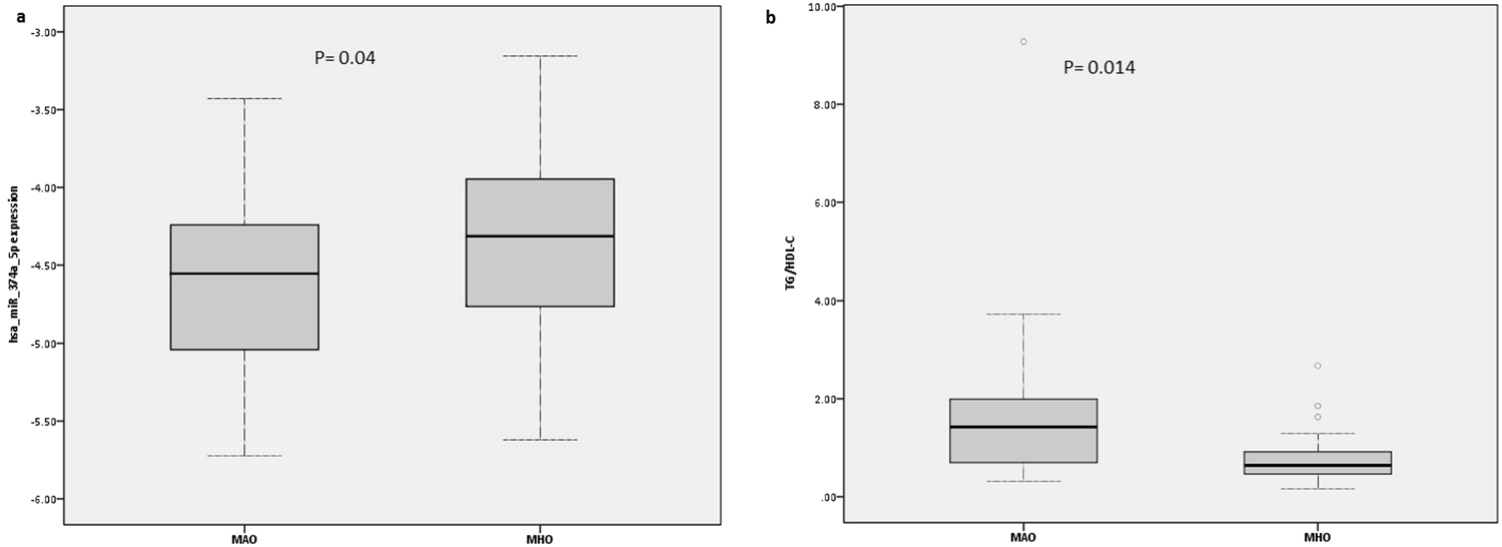
TG/HDL-C and serum miR-374a-5p levels by MHO status. (**a**) Serum miR-374a-5p levels by MHO status. (**b**) TG/HDL-C by MHO status.

An independent sample of 64 MHO and 34 MAO subjects was used to validate the 8 DEMs identified in the screening phase. In general, individuals included in the validation phase were similar in characteristic to the screening samples but were slightly younger (40.7 years vs. 42.3 years) and leaner (BMI: 38.0 Kg/m^2^ vs. 40.6 Kg/m^2^). As observed in the screening set, MHO group had better lipid, inflammatory and glycolytic profile compared to the MAO group (Table 2). Of the 8 miRNAs tested, five (miR-374a-5p, miR-26b-5p, let-7d-3p, miR-223-3p, miR-29a-3p) passed quality control (QC). Of these five, one remained significantly differentially expressed between MHO and MAO (miR-374a-5p; FC = 1.2, p = 0.04, Fig. 3, Fig. 3a).

**Table 2:** Anthropometric and clinical characteristics of participants included in the validation phase of the study.

Variable	Overall (n = 98)		MHO (n = 64)		MAO (n = 34)		P-value^¥^
	Mean (Std. Dev)	Range (Min - Max)	Mean (Std. Dev)	Range (Min - Max)	Mean (Std. Dev)	Range (Min - Max)	
AGE (Years)	40.73 (9.59)	22.00–65.00	40.05 (9.26)	23.00–61.00	42.03 (10.19)	22.00–65.00	0.3325
BMI (Kg/m^2^)	37.90 (7.49)	30.01–69.92	37.15 (6.91)	30.01–68.05	39.29 (8.43)	30.41–69.92	0.1807
WHR	0.87 (0.08)	0.64–1.06	0.85 (0.07)	0.64–1.06	0.89 (0.08)	0.73–1.03	0.0242*
SBPS (mmHg)	125.18 (14.84)	100.00–188.50	120.96 (13.75)	100.00–188.50	133.13 (13.66)	109.00–166.00	< 0.0001*
DBPS (mmHg)	79.52 (10.62)	60.00–115.00	76.77 (9.26)	60.00–110.00	84.71 (11.18)	63.00–115.00	0.0003*
Glucose (mg/dl)	85.29 (9.25)	65.00–119.00	84.08 (8.15)	65.00–97.00	87.57 (10.79)	68.00–119.00	0.0747
Log10HOMA-IR	0.35 (0.29)	−0.26–1.33	0.27 (0.21)	−0.26–0.65	0.52 (0.36)	−0.24–1.33	0.0007*
Geometric mean	**2**.**24**		**1**.**86**		**3**.**31**		
TG/HDL-C	1.05 (1.08)	0.16–9.28	0.72 (0.41)	0.16–2.67	1.68 (1.58)	0.31–9.28	0.0014*
Log_10_CRP(mg/dl)	−0.30 (0.46)	−1.40–0.57	−0.36 (0.45)	−1.40–0.50	−0.21 (0.46)	−1.22–0.57	0.1243
Geometric mean	**0**.**50**		**0**.**44**		**0**.**62**		

^¥^Student’s t-test p-values comparing mean between MHO and MAO.

*Significant p-value (p < 0.05).

### Association between miRNA-374a-5p and components of metabolic health and anthropometric markers

**Figure 4:**
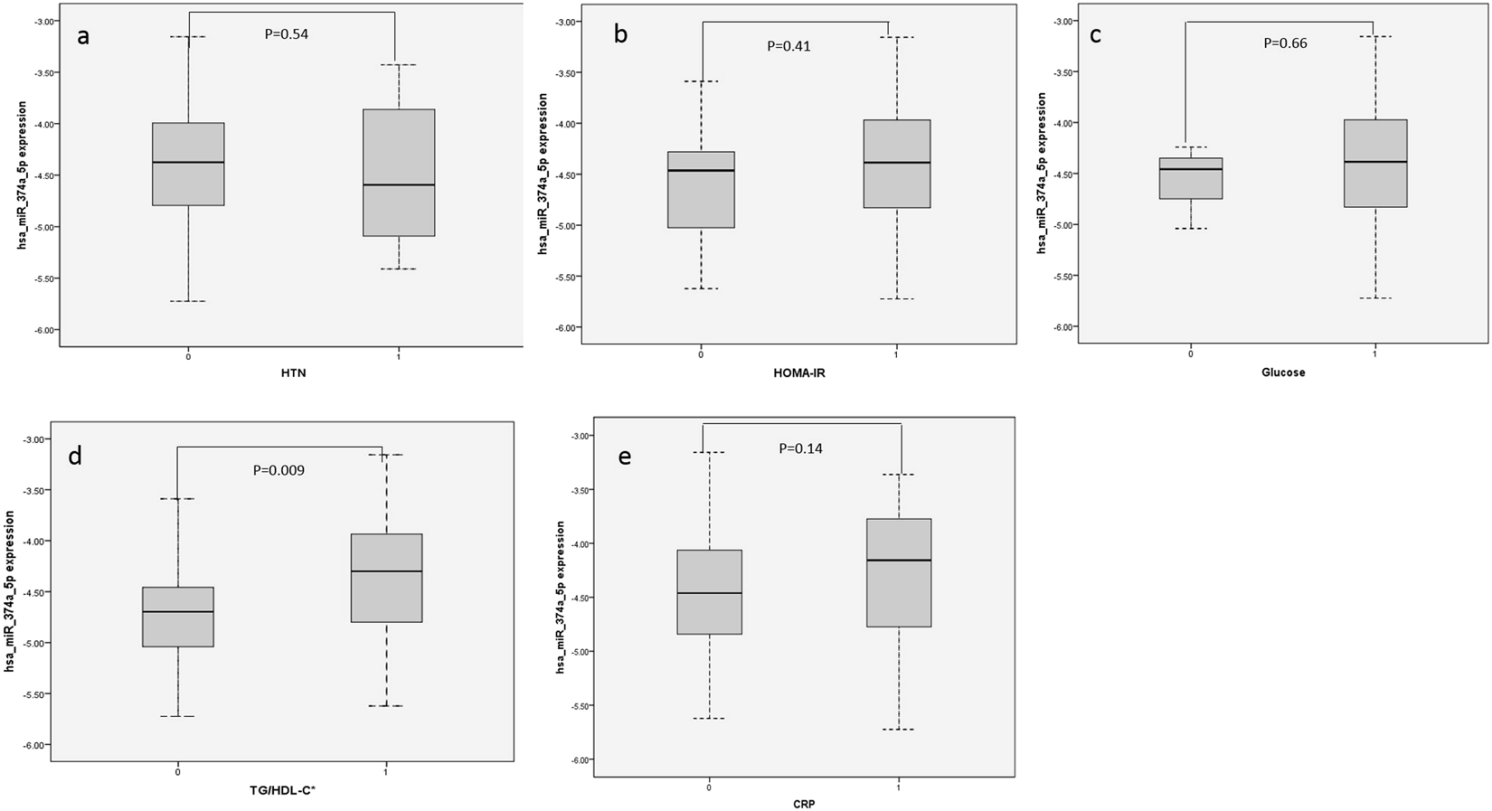
miR-374a-5p expression profile by metabolic component included in MHO definition. (**a**) HTN, hypertension; 0 = hypertensive; 1 = non-hypertensive. (**b**) HOMA-IR, 0 = HOMA-IR > 5.1; 1 = HOMA-IR <= 5.1. (**c**) Glucose, 0 = glucose > 100 mg/dl; 1 = glucose <= 100 mg/dl. (**d**) TG/HDL-C 0 = TG/HDL-C > 1.65 for male and >1.32 for female; 1 = TG/HDL-C < 1.65 for male and <1.32 for female. (**e**) CRP:0 = hsCRP > 0.3 mg/dl; 1 = hsCRP <= 0.3 mg/dl.

A Spearman’s rank-order correlation was used to evaluate the relationship between miR-374a-5p expression level and components of metabolic health in 98 obese individuals. We observed significant negative correlation between miR-374a-5p and TG/HDL-C ratio (rs = −0.224, p = 0.03). miR-374a-5p was not associated with age (p = 0.72), sex (p = 0.52) and BMI (p = 0.81). Also, miR-374a-5p was not associated with any other parameters included in the definition of MHO (hypertension p = 0.53, homeostatic model assessment-insulin resistance (HOMA-IR) p = 0.79) and CRP p = 0.67). When each of the components included in the MHO definition was coded as a binary variable, we observed no statistical difference in expression profile of miR-374-5p between the comparison groups except for TG/HDL-C. This observation suggests that the differential expression of miR-374-5p between MHO and MAO is related to changes in the TG/HDL-C ratio **(**Fig. 4). Figure 4D depicted miR-374-5p expression profile by TG/HDL-C status and showed that miR-374a-5p is upregulated in obese individuals with TG/HDL-C lower than 1.65 for males and 1.32 for females. Low to normal range TG/HDL-C ratio, a characteristic of MHO, correlated with upregulation of miR-374a-5p while high TG/HDL-C ratio correlated with downregulation. TG/HDL-C was negatively correlated with miR-374a-5p in MHO (Fig. 3b).

Given that TG/HDL-C ratio has been used as proxies of IR and inflammation, we evaluated the relationship between TG/HDL-C, HOMA-IR (an index of IR), and inflammation in this study. We observed a significant positive association between TG/HDL-C and HOMA-IR (Spearman’s Rho = 0.409, p < 0.0001)

### Putative interactions between miR-374a-5p/mRNA and biological insights

Using the miRNA target Biofilter module in IPA, we investigated the potential biological functions of miR-374a-5p in MHO. We focused this analysis on miRNA-mRNA interactions that are predicted with high confidence or are experimentally observed. MiR-374a-5p was found to target 37 mRNAs (Supplemental Table S1 and Figure S1). Targeted mRNAs included chemokines (CCL2, CCL8) and gene transcripts belonging to transmembrane proteins family (TMEMs: −185A, −267).

Core analysis showed that the 37 mRNAs targeted by miR-374a-5p were involved in inflammation pathways including interleukin-17A (IL-17A) signalling (p = 1.22 × 10^−3^), granulocytes adhesion and diapedesis (p = 7.5 × 10^−3^), and carbohydrates and lipids biosynthesis (UDP-N- acetyl glucosamine biosynthesis (p = 2.63 × 10^−3^,), oleate biosynthesis (p = 7.62 × 10^−3^) (Supplemental Figure S2**)**. The top network functions identified included *Cell-To-Cell Signalling and Interaction*, *Haematological System Development and Function*, *immune cell Trafficking* and *Tissue Morphology* which included 20 of the 37 mRNAs targeted by miR-374a-5p. The hub molecule in this network was NF-KB, a transcription factor involved in activation of pro-inflammatory processes (Supplemental Figure S3,A). The second highest ranked network among the 37 targeted mRNAs is the *Cardiovascular System Development and Function, Organismal Development* and implicated 15 of the 37 mRNAs with ERK1 and Akt as the two key hub molecules (Supplemental Figure S3,B).

**Figure 5:**
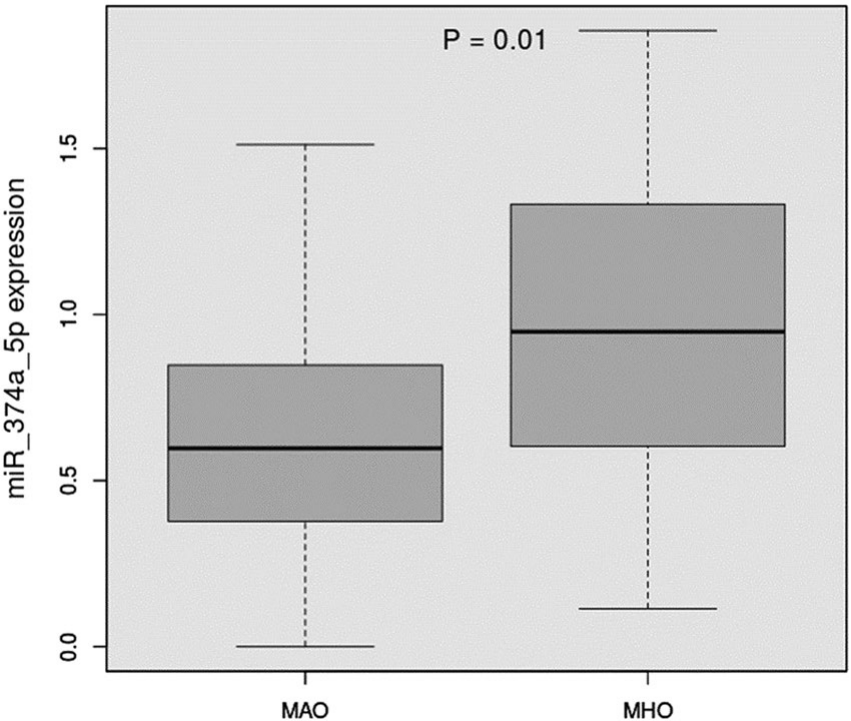
miR-374a-5p differential expression in MHO vs. MAO (independent cohort, MH-GRID).

**Figure 6:**
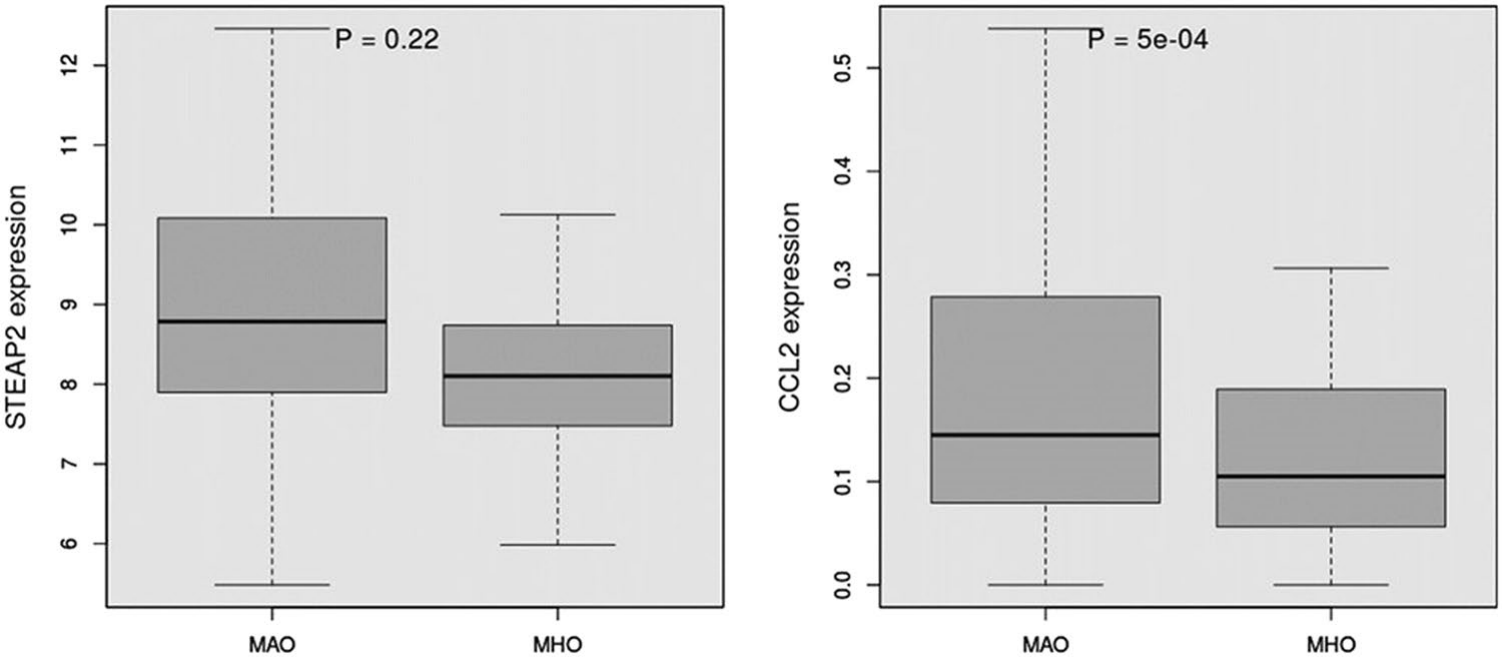
Differential expression of CCL2 and STEAP2 among MHO and MAO.

It has been shown that most of the miRNAs in circulation in obese individuals originates from adipocytes^20^. We therefore conducted analyses restricted to adipocytes. The number of targeted mRNAs was reduced to 3 (CCL2, STEAP2, and EN1). Experimental validation of the 3 targets was conducted in an independent cohort of AA obese individuals enrolled in the Minority Health Genomics and Translational Research Bio-Repository Database (MH-GRID) study (n = 80 including 25 MHOs and 55 MAOs) in which both miRNAs and RNA were sequenced. The clinical characteristics of these individuals are summarized in Supplemental Table S2. As in the other 2 cohorts, MHO individuals were younger (43.3 years vs 45.6 years) and had a better metabolic profile compared to MAO (lower CRP, TG/HDL-C, and HOMA-IR). MiR-374a-5p was also upregulated in this independent sample of MHO compared to MAO (FC = 1.34, p = 0.01) (Fig. 5). In contrast to the validation cohort however, miR-374a-5p was significantly associated with hypertension (Supplemental Figure S4). This association is likely driven by the enrichment of hypertensive in the MAO group of this cohort. While TG/HDL-C was not statistically associated with miR-374a-5p in this cohort, the upregulation trend is similar as shown in Supplemental Figure S4. Upregulation of miR-374a-5p is expected to downregulate its targets. Of the 3 mRNA targets, 2 were available for analysis, EN1 did not pass QC filters and only CCL2 was statistically significant i.e. downregulated in MHO compared to MAO (FC = 0.48, p = 0.0005) (Fig. 6).

## Discussion

The apparent paradox of obese individuals without associated comorbidities (MHO) remains an intriguing biological observation. Here, we investigated a class of molecules (miRNA) in relation to MHO phenotype. We identified eight miRNAs that were differentially expressed between MHO and MAO individuals and successfully validated one (miR-374a-5p) in two large independent samples. We observed increased levels of miR-374a-5p among the MHO compared to MAO and found that MiR-374a-5p was associated with TG/HDL-C ratio. This latter relationship in combination with an in-silico miRNA-mRNA analysis directed us to investigate the effect of miRNA 374a-5p on biomarkers (CCL2, STEAP2) upstream of the observed change in lipids.

Consistent with our findings, several miRNAs have been reported to be associated with obesity and obesity-related metabolic disorders^21,22^. Notably, miR-374a was recently observed to be differentially expressed in obese with insulin sensitivity, obese with IR, and in type 2 diabetic patients compare to lean controls in a European ancestry cohort. Furthermore, miR-374a was associated with fasting insulin^22^. Interestingly, miR-374a-5p has been observed to be differentially expressed in non-obese prediabetes and T2D patients compared to controls among Asian Indians^23^. In the same study, a gender stratified analysis revealed that the increased levels of miR-374a-5p observed in T2D patients remained significant only among the female subjects and miR-374a-5p was positively association with HOMA-IR^23^. This apparent inconsistency with the negative association we observed between miR-374a-5p and measures of IR are likely due to many factors including the different populations studied (Asian Indians vs. AAs) and perhaps more importantly, differences in the investigated phenotype: non-obese with metabolic disorders (prediabetes/diabetes in the Asian study) vs. obese metabolically healthy/unhealthy in this study). Additionally, miR-374a-5p expression direction seems to be heavily influenced by the disease or phenotype studied. For example, its levels were increased in osteosarcoma patients and aortic aneurism but decreased in patients with age-related macular degeneration^24–26^.

Changes in circulating triglycerides and HDL-C may underlie observed differential expression given that the expression patterns of miR-374a-5p in MHO vs. MAO and in low TG/HDL-C compared with high TG/HDL-C mirrors each other and no other component variable of MHO definition is associated with miR-374a-5p. However, the analysis in our independent cohort suggested that miR-374a-5p may modulate CCL2 expression, a pro-inflammatory biomarker, that is upstream in the pathway that leads to dyslipidaemia in obesity; thus, inflammation could be one of the driver of the change seen in lipid profile in obesity-related co-morbidities. Indeed, this thinking is supported by published reports that showed that deficiency in CCL2 or CCR2 results in low inflammation and protects against IR^27^. A potential mechanism underlying this relationship is increased inflammation observed in persons with high TG/HDL-C ratio. Several studies also suggested that HDL-C on its own is a modulator of systemic inflammation by displaying anti-oxidative or oxidative properties depending on the biological conditions^28^.

While reported relationships between miR-374a-5p and metabolic outcomes are mostly from observational studies with limited power to infer underlying molecular mechanisms, this study generated new avenues to investigate the functional role (s) of miR-374a-5p in insulin sensitivity via CCL2 or inflammation in general. Based on prediction, miR-374a-5p appears to regulate mRNAs that are involved in inflammation particularly transcripts in IL-17A signalling including CCL2. Activation of CCL2 is through a key transcription factor NF-KB as reflected by the functional network analysis of the mRNAs targeted by miR-374. Many of these mRNAs putatively interact with NF-KB which coordinates inflammation, ERK which modulates insulin response and inflammation, and Akt which have been implicated in the pathophysiology of metabolic disorders^29,30^. CCL2, also known as monocytes chemoattractant protein-1 (MCP-1) has been associated with IR through its ability to attract monocytes to adipose tissues and to initiate a cascade of inflammatory processes that ultimately leads to IR^31^.

There is a complex interplay between the CCL2/CCR2 axis, lipid metabolism and inflammation in the development of IR in mouse model^32^. Double mutant mouse (LDLr−/− and CCL2−/−) not only have higher plasma cholesterol and TG, but are less glucose tolerance on regular chow diet^33^. Although our study did not directly investigate LDL-C, the correlative associations observed with CCL2, HDL-C and TG suggest that they may be important players in the development of metabolic disorders. Additionally, pathways involved in lipid metabolism such as oleate biosynthesis were enriched among putative mRNAs that are targeted by miR374a-5p. Oleate appears to modulate the activity of enzymes (e.g. HMG-CoA synthase) important in cholesterol biosynthesis^34^. Taken together, these evidences suggest functional interactions between inflammation, and metabolic traits that might be modulated by epigenetic factor such as miRNAs. For example, miR-374a-5p directly targets C/EBP-β, a transcription factor that regulates acute reactant phase proteins, inflammatory cytokines while also orchestrates adipogenesis with PPAR-γ^35,36^. Our miRNA-mRNA analysis in the independent cohort suggested that upregulation of miR-374a-5p in MHO may result in downregulation of proinflammatory cytokines. The relationships between the putative mRNA and miRNA changes described in this study are correlative. Therefore, future studies should pursue functional validation of the effect of miRNA(s) on their targets in the context of obesity and more specifically in MHO using experimental procedures including miRNA mimic or inhibitor assays.

The findings of this study suggest that metabolic health in obese individuals is associated with changes in miRNA expression profile. The mechanisms involved are complex and seems to be mediated by inflammation which triggers downstream signals that appear to affect metabolic traits including lipids. Given the cross-sectional nature of this study, no causality can be inferred and the deterministic trigger of the change in expression seen in MHO compared to MAO remains elusive and require further investigation. This study attempts to reduce phenotype heterogeneity in the screening step by only including the extreme cases of MHO and MAO. This approach may have not be optimal in the identification of miRNAs that can predict MHO more globally. However, we attempted to minimize this effect in the validation phase by using a less stringent definition of MHO. Notably, findings from this study contributes to a broader understanding of the MHO phenotype by identifying a relationship between epigenetic marker (MiR-374a-5p), inflammation, and downstream pathways. These findings add a missing piece to the yet incomplete understanding of the mechanisms underlying metabolic comorbidities in some individuals with obesity.

## Methods and Experimental Design

### Subject selection and Study Design

The individuals included in this study were selected from the Howard University Family Study, a well-phenotyped cohort of AAs recruited in the Washington D.C. area to study the genetic epidemiology of complex diseases in populations of African descent as described in detail elsewhere^37^. The study was approved by the Howard University’s institutional review board (IRB) and informed consent was obtained from each participant. All experiments were performed in accordance with relevant guidelines and regulations.

A conservative approach was implemented in the selection of MHO and MAO individuals included in this study. We postulated that the use of a stringent definition of metabolic health will facilitate the identification of miRNA (s) because of the gain in specificity when compared to less stringent criteria. The selection steps are described in detail elsewhere^38^. Briefly, a combination of three distinct definitions of MHO was used: 1) the basic definition (i.e., absence of high blood pressure or no BP medication, no diabetes (fasting plasma glucose-FPG <= 126 mg/dl), and normal HDL-C levels (i.e., HDL >= 40 mg/dl for men and >= 50 mg/dl for women); 2) the modified Wildman definition (i.e. basic definition but with FPG lower to <= 100 mg/dl, absence of dyslipidaemia as measured by TG/HDL-C ratio, insulin resistance (IR) measured by HOMA-IR of <= 5.1; and 3) the most stringent definition which adds an inflammation marker (C-reactive protein, hsCRP <= 0.3 mg/dl) to all parameters included in the modified Wildman definition. All MHO subjects included in this study met these stringent inclusion criteria.

MAO subjects were required to be obese individuals who have four or all five of the following cardio-metabolic abnormalities i.e. hypertensive, no diabetes but glucose level higher than 100 mg/dl, HOMA > 5.1, TG/HDL > 1.65 for male TG/HDL > 1.32 for female and hsCRP > 0.3 mg/dl). The number of participants in the screening phase of the study was 10 MHO cases and 10 MAO controls. All twenty subjects were African-American women and their clinical and anthropometric characteristics are summarized in Table 1.

For the validation phase, a less stringent definition of metabolic health was utilized to allow for generalization of findings to a more representative cohort of MHO. Thus, MHO was defined as obese individuals with no more than one cardiometabolic abnormalities (n = 64) and MAO was defined as individuals with 2 or more cardiometabolic abnormalities (n = 34). The subjects included in the validation phase were selected from the same parent study as in the screening phase. The descriptive characteristics of the individuals included in the validation phase are summarized in Table 2.

To validate mRNAs targeted by differentially expressed miRNAs, we analysed an independent cohort of 80 obese AAs (25 MHO and 55 MAO) extracted from the MH-GRID study. The MH-GRID study is a cohort of severe hypertension and included adults between the ages of 30 and 55 and is described in detail elsewhere^39^. The study was approved by the Morehouse School of Medicine, Kaiser Permanente, Grady Health System Research Oversight Committee, and the National Institutes of Health Institutional Review Boards. All participants were appropriately consented. Metabolic health was defined using the same criteria as in the validation cohort. miRNAs and mRNAs were sequenced and quantified in peripheral blood of these individuals.

### RNA extraction from serum, cDNA synthesis and quantitative polymerase chain reaction (qPCR)

RNA was isolated from 200 µl of serum using miRCURY RNA Isolation Kit- Biofluids (Exiqon, Woburn, MA) following the manufacturer’s procedures. Briefly, proteinase and DNase were used to remove proteins and DNA from serum. To ensure reproducibility in RNA yield from serum, bacteriophage MS2 carrier RNA was added to each sample during the extraction procedure. Reverse transcription (RT) reactions were performed using Universal cDNA Synthesis Kit II (Exiqon, Woburn, MA). Negative controls (No cDNA template) were similarly processed along with the serum samples. Before using RNA samples for miRNA profiling, the yield of serum miRNAs, absence of qPCR inhibitors and haemolysis in the samples were determined using a miRNA QC PCR Panel (Exiqon, Woburn, MA).

An RT-PCR approach was used to determine serum levels of 179 human miRNAs. RNA samples from all 20 MHO and MAO individuals included in the screening phase were analysed using the 96-well Serum/Plasma Focus microRNA PCR Panel I, II (Exiqon, Woburn, MA). qPCR was performed in a 96-well thermocycler System, CFX 96 (Bio-Rad, Hercules, CA) with 40 amplification cycles using cycling parameters recommended by the manufacturer.

In the independent cohort (MH-GRID), total RNA extraction was carried out using MagMAXTM for Stabilized Blood Tubes RNA Isolation Kit as recommended by vendor (Life Technologies, Carlsbad, CA).

Total RNA samples were converted into indexed cDNA sequencing libraries using Illumina’s TruSeq sample kits (Small RNA). After PCR amplification, the final libraries were quantitated by qPCR (KAPA Library Quant Kit, KAPA Biosystems).

Read counts (expression levels) were obtained using a pipeline based on BowTie2 as alignment tool and the read count were determined using RNA-Seq by Expectation Maximization (RSEM).

MicroRNAs with less than 10 counts in more than 90% of the samples were excluded. The data was normalized, using the weighted trimmed mean of M-values (TMM) method, an optimal method for the normalization of count data.

### Data analysis

The raw data was pre-processed and analysed with *GenEx 6 Pro* Software (MultiD Analyses AB, Sweden). Briefly, after RT-PCR data was exported into *GenEX*, inter-plate calibration and quality control (QC) were performed. A cycle threshold (Ct) cut-off was used to remove reactions with low amplification efficiency, Ct > 37 was set as missing and miRNAs with a call rate < 50% were removed (n = 51). Missing data was replaced by the column mean independent of group. Data was normalised against the global mean (i.e., the average of all miRNA expressed in all samples) and then log 2 transformed for downstream analyses.

To identify differentially expressed miRNAs in this study, miRNA profiles were compared between MHO and MAO using an unpaired, two-tailed t-test. P-value ≤ 0.05 was used as cut-off to determine DEMs between the two groups. Selected DEMs were carried forward for validation in a larger independent sample of MHO and MAO individuals. The same QC pipeline was used in both the validation and screening phases except for data normalization. Validation data was normalized to the references genes miR-425-5p and miR-191-5p. The stability of these genes was tested using *geNorm* and *NormFinder*, two methods incorporated into GenEx. A miRNA is considered validated if the direction of expression is the same as in the screening dataset and the differential expression is statistically significant (p <= 0.05).

The sequenced data from the MH-GRID cohort was analysed using an R-package. We evaluated differential miRNA expression between MHO and MAO samples. DE analysis was conducted using the R library edgeR. The data was normalised, using the weighted trimmed mean of M-values (TMM) method, an optimal method for the normalization of count data. We estimated tagwise dispersion (specific dispersion level for each molecule) to respectively adjust for transcript abundance so that transcript with higher expression are not over-represented and to ensure transcripts that behave more consistently across samples are ranked higher. The dispersion estimate is included in the fitted negative-binomial model where the normalised expression is the outcome variable.

Statistical analyses of clinical and anthropometric variables were performed using *SPSS Statistics* 21.0. Continuous variables were expressed as mean +/− standard deviation. Variables that are not normally distributed were log-transformed and presented as geometric means. Student’s t-test was used to compare mean between groups. Association between miRNA expression and anthropometric and clinical markers were assessed with Spearman’s Rho (ρ) coefficient.

#### Prediction and functional analysis of miRNA targets

*Ingenuity® Pathway Analysis* (IPA®, QIAGEN Redwood City, www.qiagen.com/ingenuity) was used to search for mRNA targeted by the validated DEMs. Specifically, IPA miRNA target filter, a feature in IPA was applied. The validated DEMs in the independent cohort were uploaded into IPA and the list of putative mRNAs (Supplemental Table S1) was used to run a core analysis to identify biological pathways and network functions related to MHO.

### Data availability statement

The datasets generated and analysed during this study are available from the corresponding authors on reasonable request.

## Electronic supplementary material

Supplementary Information
