# Supplementary material for: Circulating MiR-374a-5p is a potential modulator of the inflammatory process in obesity
Source: Sci Rep. 2018 May 16;8:7680. doi: 10.1038/s41598-018-26065-5 (PMC5955981; doi:10.1038/s41598-018-26065-5)

Circulating MiR-374a-5p is a potential modulator of the inflammatory process in obesity

Ayo P Doumatey[1,2*], William J He [1,2,3], Amadou Gaye[1], Lin Lei [1,2], Jie Zhou[1,2], Gary Gibbons[1,4] , Adebowale Adeyemo[1,2], and Charles N Rotimi[1,2*]

[1]*Metabolic, Cardiovascular and Inflammatory Disease Genomics Branch, National Human Genome Research Institute, Bethesda, MD USA*

[2] *The Center for Research on Genomics and Global Health, National Human Genome Research Institute, National Institutes of Health, Bethesda, Maryland*

[3]*Johns Hopkins University, Krieger School of Arts and Sciences, Baltimore, Maryland*

[4]*National Heart, Lung, and Blood Institute, National Institutes of Health, Bethesda, MD USA*

Table S1. List of mRNAs targeted by miR374a-5p

miRNA	Source	Confidence	Targeted mRNA
hsa-miR-374a-5p	TargetScan Human	High (predicted)	ANKRA2
hsa-miR-374a-5p	TargetScan Human	High (predicted)	ANXA1
hsa-miR-374a-5p	TargetScan Human	High (predicted)	ATF7
hsa-miR-374a-5p	TargetScan Human	High (predicted)	BOLA3
hsa-miR-374a-5p	TargetScan Human	High (predicted)	C1orf141
hsa-miR-374a-5p	TargetScan Human	High (predicted)	CBWD1
hsa-miR-374a-5p	TargetScan Human	High (predicted)	CBWD5
hsa-miR-374a-5p	TargetScan Human	High (predicted)	CCDC179
hsa-miR-374a-5p	TargetScan Human	High (predicted)	CCL2
hsa-miR-374a-5p	TargetScan Human	High (predicted)	CCL8
hsa-miR-374a-5p	TargetScan Human	High (predicted)	COMMD8
hsa-miR-374a-5p	TargetScan Human	High (predicted)	DPY19L4
hsa-miR-374a-5p	TargetScan Human	High (predicted)	EN1
hsa-miR-374a-5p	TargetScan Human	High (predicted)	FUT9
hsa-miR-374a-5p	TargetScan Human	High (predicted)	FZD3
hsa-miR-374a-5p	TargetScan Human	High (predicted)	GRXCR1
hsa-miR-374a-5p	TargetScan Human	High (predicted)	HIBADH
hsa-miR-374a-5p	TargetScan Human	High (predicted)	HSBP1
hsa-miR-374a-5p	TargetScan Human	High (predicted)	KCTD19
hsa-miR-374a-5p	TargetScan Human	High (predicted)	LINC01549
hsa-miR-374a-5p	TargetScan Human	High (predicted)	LOC107986898
hsa-miR-374a-5p	TargetScan Human	High (predicted)	MBLAC2
hsa-miR-374a-5p	TargetScan Human	High (predicted)	NCK1
hsa-miR-374a-5p	TargetScan Human	High (predicted)	NME5
hsa-miR-374a-5p	TargetScan Human	High (predicted)	NPPC
hsa-miR-374a-5p	TargetScan Human	High (predicted)	NTF3
hsa-miR-374a-5p	TargetScan Human	High (predicted)	PLAC8
hsa-miR-374a-5p	TargetScan Human	High (predicted)	PPM1B
hsa-miR-374a-5p	TargetScan Human	High (predicted)	PRR16
hsa-miR-374a-5p	TargetScan Human	High (predicted)	PUS10
hsa-miR-374a-5p	TargetScan Human	High (predicted)	SCML1
hsa-miR-374a-5p	TargetScan Human	High (predicted)	SMKR1
hsa-miR-374a-5p	TargetScan Human	High (predicted)	STEAP2
hsa-miR-374a-5p	TargetScan Human	High (predicted)	TMEM185A
hsa-miR-374a-5p	TargetScan Human	High (predicted)	TMEM267
hsa-miR-374a-5p	TargetScan Human	High (predicted)	TTC32
hsa-miR-374a-5p	TargetScan Human	High (predicted)	ZBBX

Figure S1. miR-374a-5p interacts with 37 mRNAs (IPA analysis)

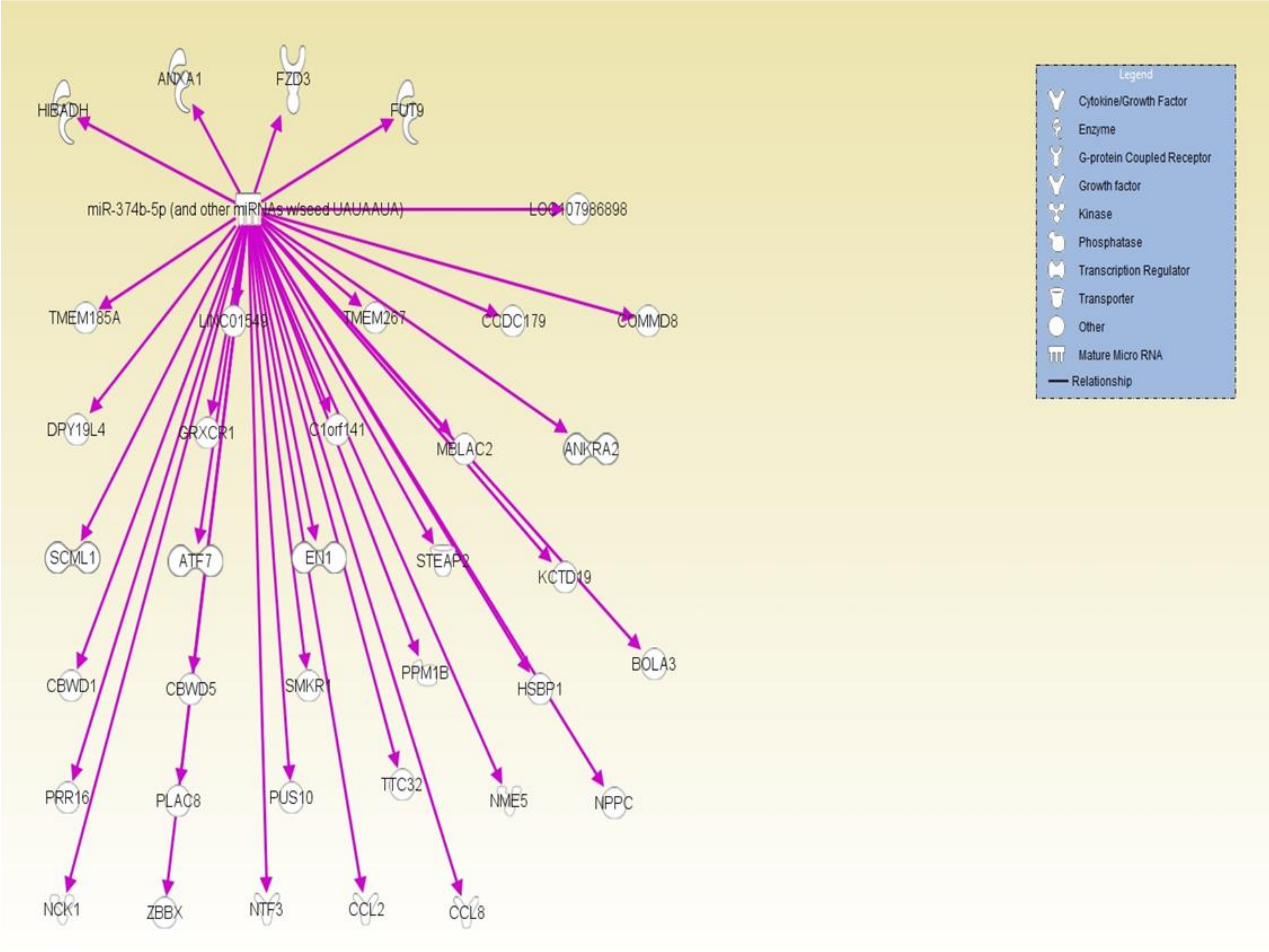

© 2000-2017 QIAGEN. All rights reserved.

Figure S2: Canonical Pathways enriched among the 37 mRNAs regulated by miR-374a-5p

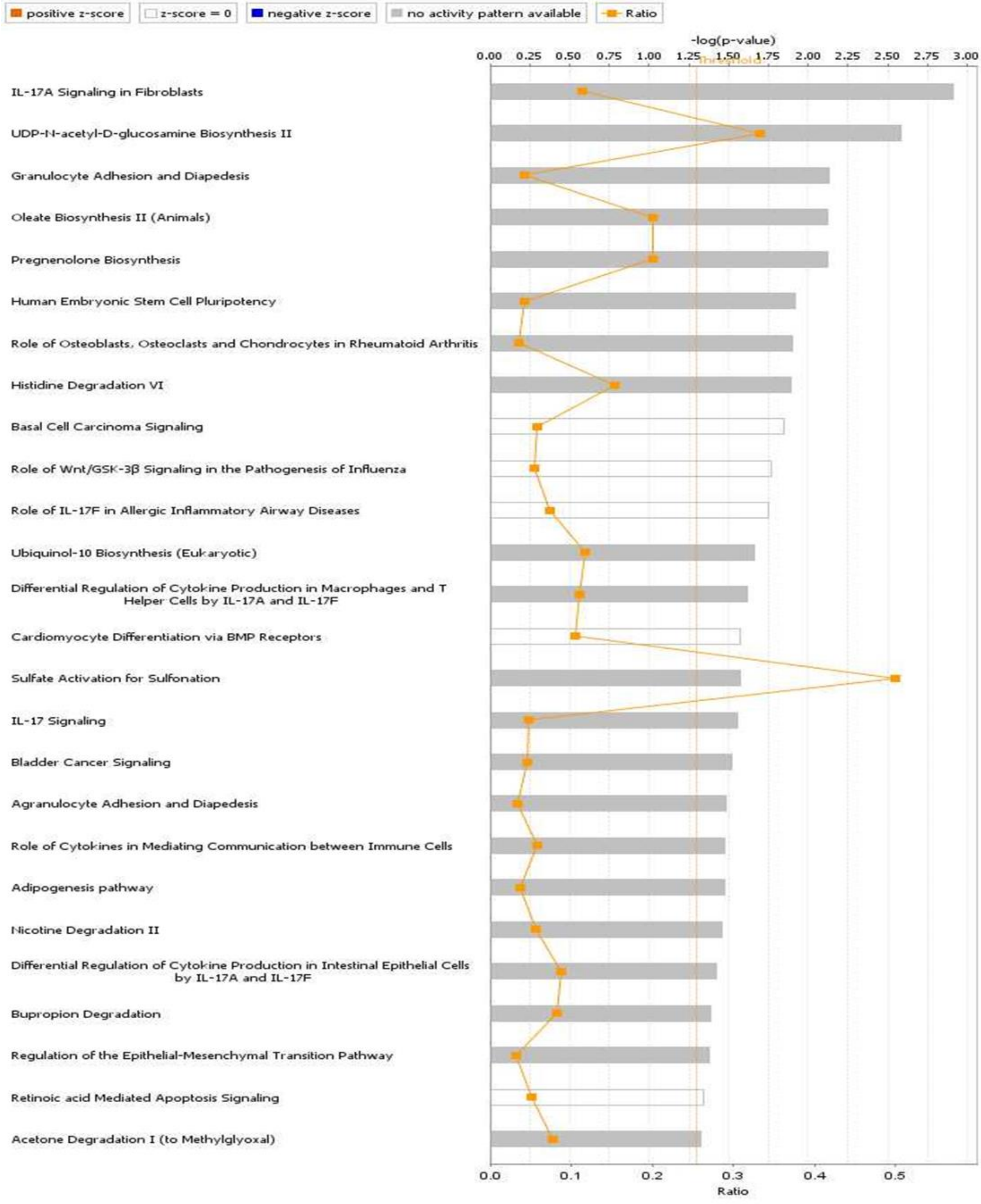

© 2000-2017 QIAGEN. All rights reserved.

Figure S3. Top networks associated with the 37 mRNAs targeted by miR-374a-5p

A

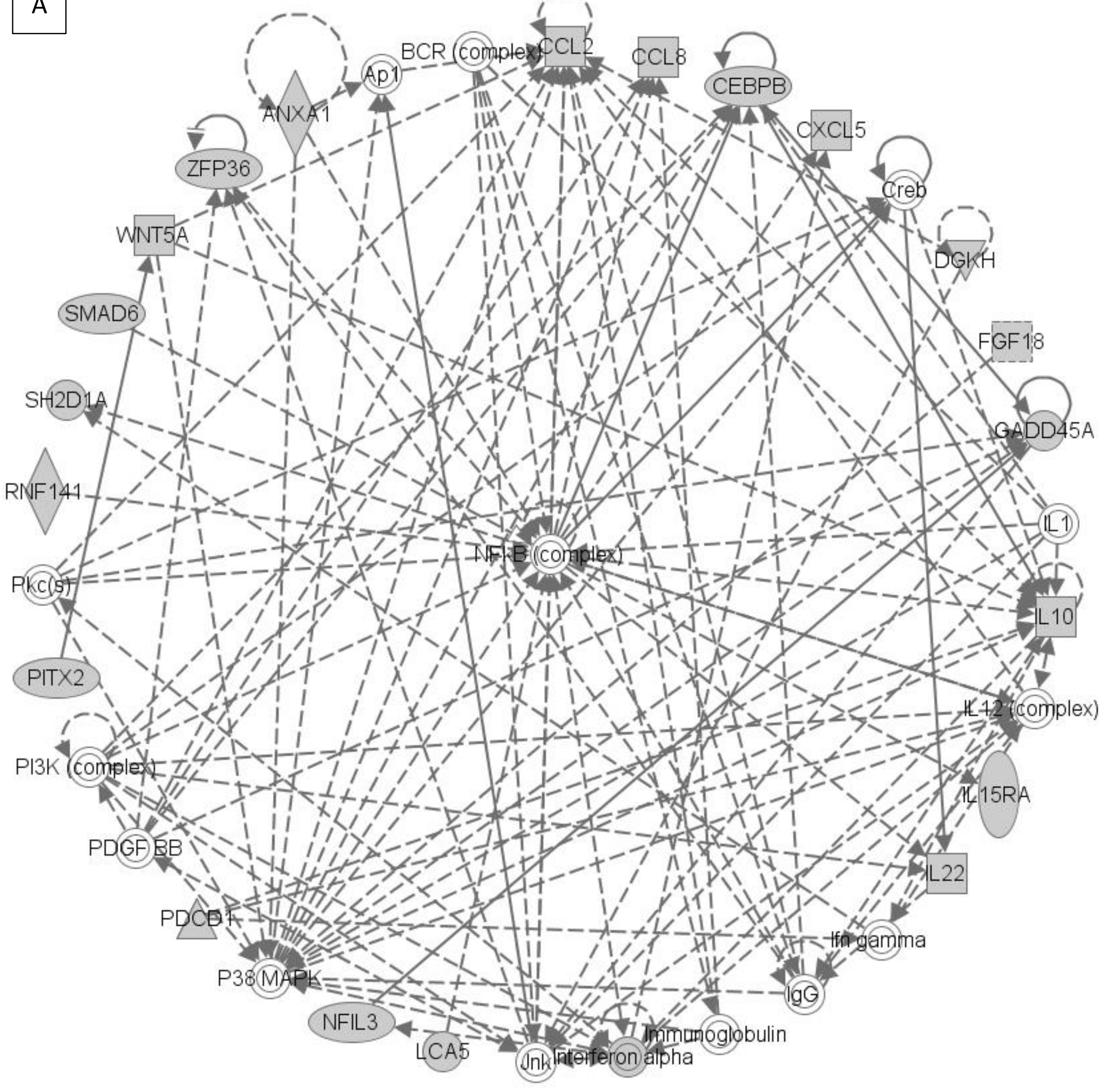

© 2000-2017 QIAGEN. All rights reserved.

Cell-To-Cell Signaling and Interaction, Hematological System Development and Function, immune cell Trafficking (20 focus molecules)

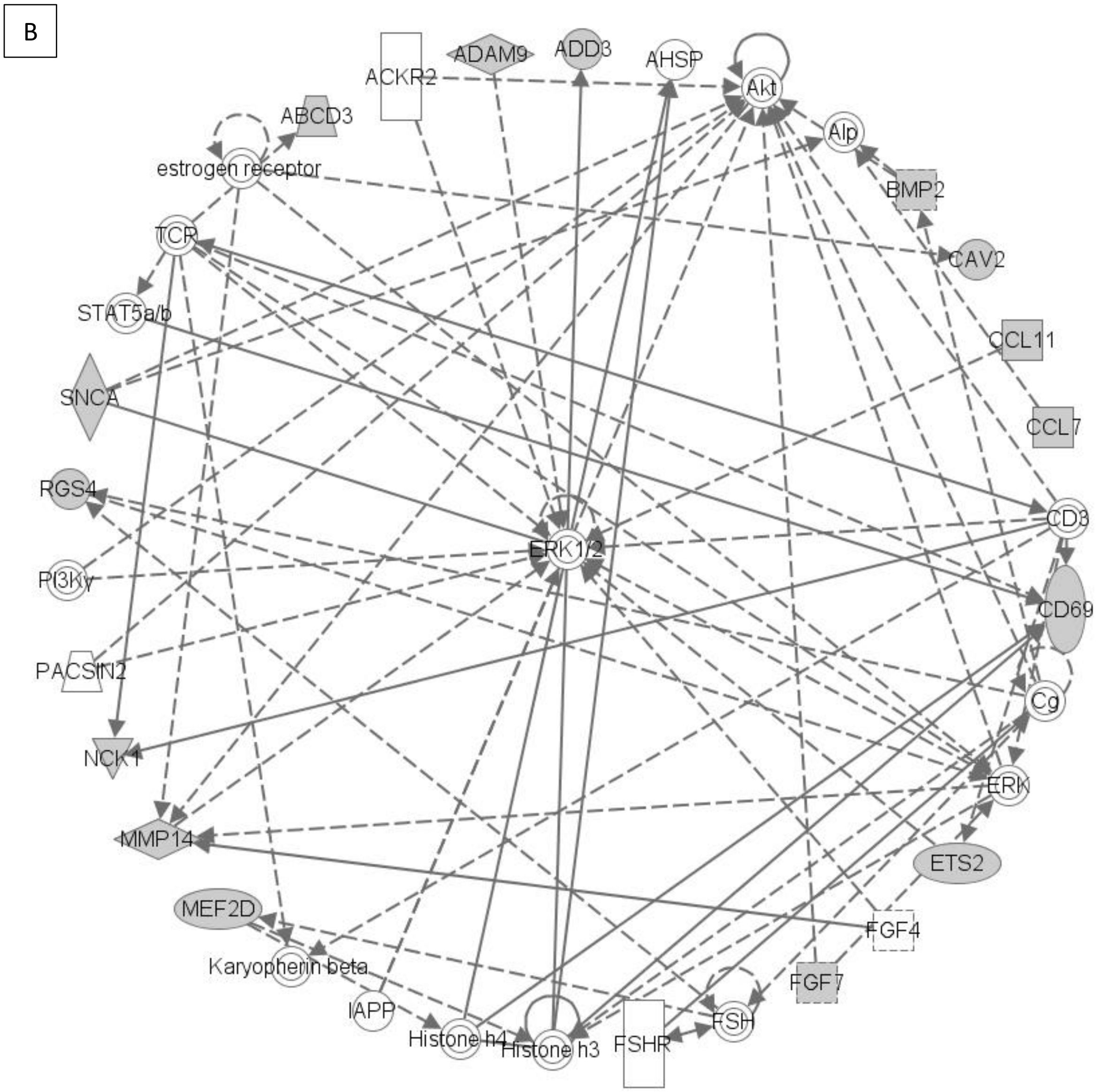

© 2000-2017 QIAGEN. All rights reserved.

Tissue Morphology, Cardiovascular System Development and Function, Organismal Development (15 focus molecules)

Table S2: Anthropometric and clinical characteristics of the MH-GRID cohort

Variables	All Samples (n=80)	Range (All Samples)	MHO(n=25)	Range(MHO)	MAO(n=55)	Range(MAO)	p.value
Age (years)	44.85 ± 6.76	[30,55]	43.32 ± 6.72	[33,54]	45.55 ± 6.73	[30,55]	0.18
BMI (kg/m2)	37.93 ± 7.24	[30.05,69.2]	35.59 ± 5.34	[30.22,51.34]	38.99 ± 7.77	[30.05,69.2]	0.03
WHR	1.11 ± 0.1	[0.95,1.39]	1.11 ± 0.09	[1.01,1.31]	1.11 ± 0.1	[0.95,1.39]	0.98
SBP (mmHg)	117.9 ± 14.01	[90,159]	114.2 ± 6.16	[100,126]	119.58 ± 16.16	[90,159]	0.03
DBP (mmHg)	77.04 ± 9.42	[58,106]	72.84 ± 5.59	[58,83]	78.95 ± 10.2	[62,106]	9.36E-04
Glucose (mg/dl)	92.35 ± 9.22	[75,122]	87.92 ± 6.53	[77,99]	94.36 ± 9.6	[75,122]	8.26E-04
log10(HOMA-IR)	4.16 ± 3.17	[0.3,14.65]	2.07 ± 1.12	[0.3,4.56]	5.35 ± 3.34	[0.94,14.65]	1.67E-07
TG/HDL	2.23 ± 1.3	[0.52,6.96]	1.48 ± 0.77	[0.52,3.93]	2.58 ± 1.36	[0.85,6.96]	1.81E-05
log10(CRP) (mg/dl)	0.5 ± 0.34	[-0.52,1.17]	0.24 ± 0.28	[-0.52,0.66]	0.65 ± 0.27	[-0.03,1.17]	4.65E-07

Figure S4. miR-374a-5p expression profile by metabolic component included in MHO definition (MH-GRID cohort)

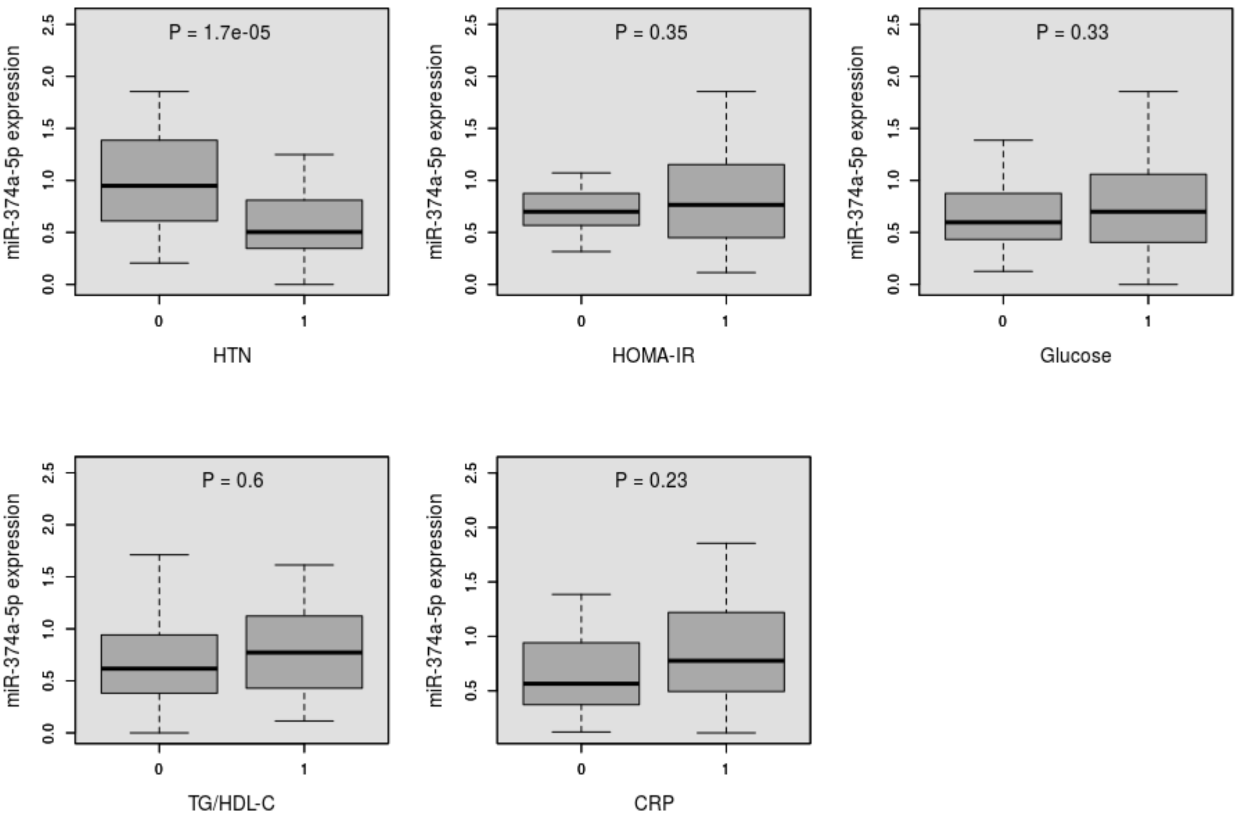

Supplement: Supplementary file 1 — Supplementary Information [file 41598_2018_26065_MOESM1_ESM.pdf]
